# Ergothioneine and its congeners: anti-ageing mechanisms and pharmacophore biosynthesis

**DOI:** 10.1093/procel/pwad048

**Published:** 2023-08-10

**Authors:** Li Chen, Liping Zhang, Xujun Ye, Zixin Deng, Changming Zhao

**Affiliations:** Department of Geriatrics, Zhongnan Hospital of Wuhan University, Wuhan University, Wuhan 430072, China; Key Laboratory of Combinatory Biosynthesis and Drug Discovery, School of Pharmaceutical Sciences, Ministry of Education, Wuhan University, Wuhan 430072, China; Key Laboratory of Combinatory Biosynthesis and Drug Discovery, School of Pharmaceutical Sciences, Ministry of Education, Wuhan University, Wuhan 430072, China; Department of Geriatrics, Zhongnan Hospital of Wuhan University, Wuhan University, Wuhan 430072, China; Department of Geriatrics, Zhongnan Hospital of Wuhan University, Wuhan University, Wuhan 430072, China; Key Laboratory of Combinatory Biosynthesis and Drug Discovery, School of Pharmaceutical Sciences, Ministry of Education, Wuhan University, Wuhan 430072, China; Department of Geriatrics, Zhongnan Hospital of Wuhan University, Wuhan University, Wuhan 430072, China; Key Laboratory of Combinatory Biosynthesis and Drug Discovery, School of Pharmaceutical Sciences, Ministry of Education, Wuhan University, Wuhan 430072, China

**Keywords:** Ergothioneine, antioxidant, anti-ageing, longevity, biosynthesis, enzymology

## Abstract

Ergothioneine, Ovothiol, and Selenoneine are sulfur/selenium-containing histidine-derived natural products widely distributed across different organisms. They exhibit significant antioxidant properties, making them as potential lead compounds for promoting health. Increasing evidence suggests that Ergothioneine is positively correlated with healthy ageing and longevity. The mechanisms underlying Ergothioneine's regulation of the ageing process at cellular and molecular levels are beginning to be understood. In this review, we provide an in-depth and extensive coverage of the anti-ageing studies on Ergothioneine and discuss its possible intracellular targeting pathways. In addition, we highlight the recent efforts in elucidating the biosynthetic details for Ergothioneine, Ovothiol, and Selenoneine, with a particular focus on the study of their pharmacophore-forming enzymology.

## Introduction

Ergothioneine (EGT), a sulfur-containing histidine derivative, has received extensive attention as an efficient antioxidant for decades. Among the multitude of naturally occurring antioxidants, Ergothioneine is both abundant and unique. It is produced by a wide variety of species, including editable fungi and some prokaryotes. Mammals cannot synthesize Ergothioneine, but possess a highly specific organic cation transporter OCTN1 (now known as solute carrier family 22 member 4, SLC22A4) allowing for efficient absorption of Ergothioneine from one’s daily diet, leading to accumulation of Ergothioneine in tissues and organs of humans and other animals (Gründemann et al., 2005; Nikodemus et al., 2011). An ABC transporter specific for Ergothioneine has recently been discovered in gastrointestinal microbes. This indicates dietary Ergothioneine was competitively absorbed and metabolized by gut microbes, which may potentially affect its absorption in humans (Dumitrescu et al., 2022; Zhang et al., 2022).

Many literatures provide an overview of Ergothioneine’s diverse cytoprotective properties, with particular emphasis on its antioxidant activity (Halliwell et al., 2018; Borodina et al., 2020; Cheah and Halliwell, 2020; Fu and Shen, 2022). Ergothioneine scavenges reactive oxygen species (ROS) and reactive nitrogen species (RNS), which are known to damage DNA, proteins and lipids (Stoffels et al., 2017; Halliwell et al., 2018). Ergothioneine chelates metal cations such as Cu^2+^/Cu^+^ so as to inhibit the production of ROS (De Luna et al., 2013). On the other hand, it activates antioxidation enzymes via modulating cellular antioxidant defense systems (Colognato et al., 2006; Hseu et al., 2015; Dare et al., 2021). The antioxidation properties, together with its ultraviolet/infrared (UV/IR) radiation protective efficacy (Obayashi et al., 2005; Bazela et al., 2014; Hseu et al., 2015) and anti-inflammatory activities (Asahi et al., 2016; Cheah et al., 2017), render Ergothioneine an attractive choice for cosmetics, dietary supplements, and other applications.

During the last 2 decades, the anti-ageing research has been brought to the forefront of science. Evidence from model animal and human studies increasingly suggests that Ergothioneine is associated with healthy aging and actives against age-related diseases. Ergothioneine was found to have a positive age-prolonging effect in *Drosophila melanogaster*, and *Caenorhabditis elegans* exhibited a shorter lifespan when the Ergothioneine transporter was knocked out (Cheah et al., 2013; Pan et al., 2022). Low levels of Ergothioneine in blood and plasma are associated with frailty in the elderly population (Kameda et al., 2020; Teruya et al., 2021) as well as several age-related diseases, including neurodegenerative diseases (Jang et al., 2004; Yang et al., 2012; Cheah et al., 2016; Wu et al., 2021), chronic inflammation (Shimizu et al., 2015; Cheah et al., 2017), cardiovascular diseases (Smith et al., 2020; Lam-Sidun et al., 2021), and diabetes (Dare et al., 2021). On the other hand, the risk of several of these diseases is reduced when mushrooms, a major source of Ergothioneine, are consumed in increased quantities (Zhang et al., 2017; Ba et al., 2022), although the protective effects of Ergothioneine on the development of those diseases have not been established. Because of its anti-ageing effect, Ergothioneine was described as one of the “longevity vitamins” by Bruce Ames (2018). These findings, combined with people’s pressing requirement for healthy ageing, call for further research on Ergothioneine’s role in longevity and anti-senescence control, especially its molecular mechanisms underlying anti-ageing properties (Apparoo et al., 2022). In this review, we provide an overview of the antioxidant characteristics of Ergothioneine, which functions as both a ROS scavenger and an antioxidant defense regulator in modulating the Kelch-like ECH-associated protein 1 (KEAP1) and nuclear factor erythroid 2-related factor 2 (NRF2) signaling pathway. Furthermore, we highlight the possible involvement of Ergothioneine in ameliorating the development of genomic instability and ageing-associated epigenetic alterations, which are two of the primary hallmarks of ageing (López-Otín et al., 2013, 2023).

**Figure 1.**
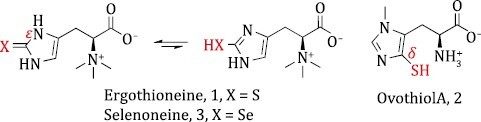
**Chemical structures of Ergothioneine, Ovothiol, and Selenoneine**. The sulfur atom is respectively installed at the ε- and δ-carbon of the imidazole ring in Ergothioneine and Ovothiol, while it is a selenium attached at the ε-position in Selenoneine.

In addition to Ergothioneine’s remarkable anti-ageing properties, the biosynthesis of Ergothioneine and its congeners, Ovothiol and Selenoneine (Fig. 1), has attracted widespread attention. This is because nature has evolved unique biosynthetic strategies for the formation of their pharmacophores, including the thiol groups in Ergothioneine and Ovothiol, as well as the selenol group of Selenoneine, a selenium analogue of Ergothioneine. The non-heme iron enzyme, sulfoxide synthase, catalyzes unusual oxidative carbon-sulfur bond formation in the biosynthesis of Ergothioneine and Ovothiol. Strong efforts have been made to elucidate the catalytic mechanisms of these enzymes, including enzyme biochemistry, steady-state kinetics, protein crystallography, unnatural amino acid incorporation, and computational modeling. We herein highlight representative enzymatic models of oxygen-dependent sulfoxide synthases, which are the most popular enzymes responsible for C–S bond formation in Ergothioneine and Ovothiol biosynthesis. We summarize the recent progress on Ergothioneine oxygen-independent formation and Selenoneine biosynthesis as well.

## Ergothioneine and its proposed anti-ageing mechanisms

### A direct antioxidant or a regulator of the antioxidant defense system

**Figure 2.**
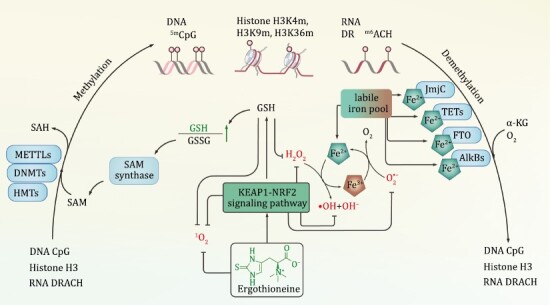
**Ergothioneine’s potential impacts on epigenetic methylation and demethylation via influencing cellular redox homeostasis**. Ergothioneine targets cellular ROS, including O_2_·^−^, H_2_O_2_, ·OH, and ^1^O_2_, in the manner of both a self-sacrificing antioxidant and a regulator of cellular antioxidant defense system, which modulates the cellular GSH redox state. When GSH levels are sufficiently high, it promotes the maximal activity of SAM synthase. Epigenetic methyltransferases, such as METTLs, DNMTs, and HMTs, utilize SAM as a substrate and their catalytic activity may be therefore influenced by Ergothioneine. On the other hand, cellular unliganded iron promotes hydroxyl radical production through the Fenton and Haber–Weiss reaction. Those iron from labile iron pool are also the essential co-factor of epigenetic demethyltransferases, including JmjC, TETs, FTO, and AlkBs. As ergothioneine scavenges ROS and maintains the labile iron pool, it ensures the activity of enzymes involved in epigenetic demethylation.

As an antioxidant, Ergothioneine was found to be an effective ROS scavenger *in vitro.* Ergothioneine reacts rapidly with singlet oxygen (^1^O_2_) (Stoffels et al., 2017), and scavenges other ROS species including superoxide radicals (O_2_·^−^), hydrogen peroxides (H_2_O_2_), hydroxyl radicals (·OH), peroxynitrite (ONOO^−^) and hypochlorite (ClO^−^) (Fig. 2) (Akanmu et al., 1991; Aruoma et al., 1997; Servillo et al., 2015, 2017; Stoffels et al., 2017; Oumari et al., 2019; Ando and Morimitsu, 2021). Even at nanomolar levels, Ergothioneine has impressive cellular antioxidant properties (Hseu et al., 2015), although its reported redox potential (*E*^0^ = −0.06 V) is not as low as that of the classic reductants, such as glutathione (GSH) (*E*^0^ = −0.24 V), and certain antioxidant vitamins (Yadan, 2022; Hondal, 2023). As reviewed by Halliwell et al., in living cells, Ergothioneine may not principally react to ROS the same way as primary antioxidants do. It becomes important only when primary antioxidants, such as GSH, are exhausted during oxidative stress (Paul, 2022; Halliwell et al., 2023). In a review about antioxidant therapy, it has been claimed that antioxidant enzymes provide the predominant antioxidant defense because they react 10^3^–10^6^ of times more rapidly with ROS than small molecule antioxidants do (Forman and Zhang, 2021). Therefore, one hypothesis is that Ergothioneine may function as a regulator of antioxidant defense system more than a direct antioxidant.

**Figure 3.**
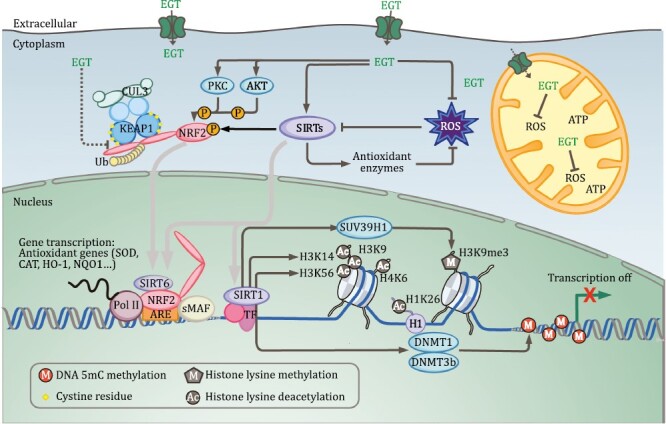
**Interaction of Ergothioneine with KEAP1–NRF2 and Sirtuin pathways**. Ergothioneine regulates KEAP1–NRF2 complex, as well as SIRT1 and SIRT6 expression levels, to upregulate antioxidant genes, such as superoxide dismutase (SOD), catalase (CAT), heme oxygenase-1 (HO-1) and NAD(P)H:quinone oxidoreductase (NQO1). SIRT1 deacetylates NRF2, histone H1, H3, and H4, and modulates the activity of certain epigenetic enzymes, including methyltransferase SUV39H1 for histone H3K9me3 trimethylation, DNMT1 and DNMT3b for DNA 5mC methylation.

Several studies reported that Ergothioneine plays its role of antioxidation and anti-ageing by interacting with intercellular signaling cascades *in vivo*, such as the KEAP1 and NRF2 signaling pathway (Hseu et al., 2015, 2020; Dare et al., 2021; Salama et al., 2021). As part of the cellular antioxidant defense systems, the KEAP1–NRF2 signaling pathway plays critical roles in maintaining redox balance, defending against oxidative stress and inflammation (Surh, 2003). Under normal conditions, NRF2 is bound to Cullin3 (CUL3) and KEAP1 for its proteasomal degradation, ensuring the low abundance of cellular NRF2 (Fig. 3). But when exposed to oxidative stress, KEAP1/CUL3 polyubiquitination is hindered, thereby leading NRF2 to be released and then translocated to the nucleus, where NRF2 binds to the Antioxidant Response Element (ARE) and triggers the activation of a variety of antioxidant genes (Ganesh Yerra et al., 2013). Accumulating evidences have shown the significant contributions of the KEAP1–NRF2 system to the prevention and attenuation of ageing and ageing-related diseases (Zhang et al., 2015; Hiebert et al., 2018; Matsumaru and Motohashi, 2021).

Experimental evidences have indicated that Ergothioneine regulates the KEAP1–NRF2 pathway in a dose-dependent manner, resulting in the upregulation of downstream antioxidant genes, including heme oxygenase-1 (HO-1), NAD(P)H: quinone oxidoreductase (NQO1), and superoxide dismutase (SOD) and catalase (CAT) (Hseu et al., 2015, 2020; Dare et al., 2021). NRF2 can detach itself from the KEAP1–NRF2 complex by self-modifications, for example, phosphorylation of its serine or threonine residues by cellular kinases, and (or) deacetylation by Sirtuins (Liu et al., 2021). Studies indicated that upon Ergothioneine treatment the protein levels of phosphatidylinositol 3-kinase (PI3K), serine/threonine kinase (AKT) and protein kinase C (PKC) are elevated. Additionally, it has also been revealed that NRF2 translocation was mediated by PI3K/AKT and PKC signaling pathways (Hseu et al., 2015, 2020). An *in silico* work indicated that Ergothioneine is an allosteric effector of NRF2, suggesting a direct interaction of Ergothioneine with NRF2 (Dare et al., 2022). Alternatively, Ergothioneine influences the KEAP1–NRF2 pathway possibly via its interaction with KEAP1, a thiol-rich protein. The abundant cysteine residue thiol groups presented on KEAP1 surface are specific sensors, which can interact with both redox-disrupting stimuli (such as electrophiles and ROS) (Zhang and Hannink, 2003), and endogenous or exogenous metabolites (e.g., Itaconate and Sulforaphane), thus resulting in the deactivation of KEAP1 and nuclear accumulation of NRF2 (Dinkova-Kostova et al., 2002; Hong et al., 2005; Mills et al., 2018). As for Ergothioneine, it is possible that it activates the KEAP1 cysteine sensors, leading to the dissociation of KEAP1–NRF2 complex and activation of antioxidant genes, however, this kind of possibility remains to be examined. It is worth investigating the interaction mechanisms between Ergothioneine and KEAP1–NRF2 signaling pathway.

### Ergothioneine facilitates genome stability

Several hallmarks of human ageing have been identified and included as a significant part of the fundamental criteria for longevity intervention discoveries (López-Otín et al., 2013, 2023; Partridge et al., 2020). Among them, genomic instability, epigenetic alterations, telomere attrition, loss of proteostasis, and disabled macroautophagy are suggested as primary hallmarks. We explore Ergothioneine’s contributions toward genome stability as well as epigenetic modifications in this review.

DNA damage, largely in consequence of oxidative stress, plays a critical role in the ageing process and influences several key aspects of the ageing phenotype (Hasty et al., 2003; Kujoth et al., 2005; Schumacher et al., 2021). The integrity and stability of both nuclear DNA and mitochondrial DNA (mtDNA) are continuously declined due to exogenous damage, including chemicals and UV/IR irradiation, as well as endogenous damage, such as ROS and RNS (Hoeijmakers, 2009). As an effective antioxidant, Ergothioneine was shown to prevent DNA damage induced by ROS and RNS (Colognato et al., 2006; Markova et al., 2009; Paul and Snyder, 2010; Zhu et al., 2011; Hseu et al., 2015). In addition, Ergothioneine was known as a physiological protectant against UV rays-induced ROS generation and damage since it can absorb light in the UV range directly, as well as enable DNA repair in UV-irradiated cells (Carlsson et al., 1974; Markova et al., 2009). Pre-treatment of HaCaT cells with Ergothioneine suppresses the intercellular ROS level induced by UVA and protects DNA against oxidative damage (Hseu et al., 2015). Cells lacking the Ergothioneine transporter exhibited an increased level of DNA damage (Paul and Snyder, 2010).

Mitochondria, the energy factory of our body, produce the vast majority of cellular ATP, as well as ~90% of ROS, such as superoxide radicals and hydrogen peroxide. In the process of ageing, one of the main reasons that mtDNA shows a higher damage level than nuclear DNA is the proximity of ROS sources (Balaban et al., 2005). It has been reported that Ergothioneine may also reduce ROS production and inhibit oxidative damage to mtDNA (Paul and Snyder, 2010). In another study, Ergothioneine reduces mitochondria specific hydrogen peroxide production in the rat kidney (Williamson et al., 2020). Growing evidences suggested that OCTN1 is also present in mitochondria: An increased radioactivity was detected in the mitochondria of rat liver after injecting ^3^H labeled-Ergothioneine in rat (Kawano et al., 1982). Upon exposure to Ergothioneine, a significant accumulation of Ergothioneine in mitochondria was observed in both cells and tissues, as reviewed by Halliwell et al. (2023), from their unpublished data. However, at present, OCTN1’s mitochondrial location remains controversial due to an absence of conclusive evidence (Gründemann et al., 2022). While it would make sense for the Ergothioneine transporter to be found in mitochondria, more direct evidence may be needed.

### Ergothioneine’s potential impacts on epigenetic methylation/demethylation

In addition to genomic instability, epigenetic alteration, including DNA and RNA methylation and histone modification, is another layer of age-related changes that harm the basic functions of cells and increases the risk of age-related diseases (Rando and Chang, 2012; López-Otín et al., 2013; Sen et al., 2016). C5 methylation of cytosine in DNA CpG dinucleotides (5mC) is the most abundant type of DNA epigenetic marker, and its pattern is altered with age (Horvath, 2013; Horvath and Raj, 2018). Post-translational modifications of histone, including methylation and acetylation, are crucial to chromatin function and vary with age. Many naturally occurring antioxidants have been found to exert their activity via epigenetic mechanisms, such as reversal of altered DNA methylation patterns (Kaufman-Szymczyk et al., 2015; Arora et al., 2019; Beetch et al., 2020). As reviewed by Hitchler et al., the intracellular redox chemistry has the potential to generate significant alterations in the epigenetic landscape through GSH and iron ions, which curtail the accessibility of epigenetic co-factors including *S*-adenosylmethionine (SAM), α-ketoglutarate, ascorbate, and nicotine adenine dinucleotide (NAD^+^) (Hitchler and Domann, 2021). Considering the close relationship between cellular redox metabolites and epigenetic modifications, it can be inferred that antioxidants and epigenomes are also potentially linked. The beneficial effects of Ergothioneine as a potent antioxidant and anti-ageing agent might also be mediated through epigenetic modifications on DNA, RNA, and Histone, although this possibility remains to be fully investigated.

#### Epigenetic methylation

DNA methyltransferases (DNMTs), using SAM as the methyl donor, are responsible for transferring methyl groups to the 5-position of cytosine residue in DNA to generate 5mC (Fig. 2). SAM availability is influenced by cellular GSH, which has been reported to play an essential role in the regulation of epigenetic methylations (Lertratanangkoon et al., 1996, 1997). First, GSH and SAM share a homocysteine intermediate in the *de novo* synthetic pathway, Ergothioneine therefore would contribute to SAM availability by affecting GSH level. Over recent years, growing evidence has indicated a link between Ergothioneine and GSH function. Ergothioneine eliminates ROS through direct or indirect mechanisms thereby modulating the cellular GSH redox state (Jacob, 2006; Servillo et al., 2015; Oumari et al., 2019; Hartmann et al., 2023). Second, GSH redox status influences the activity of SAM synthase (also named methionine adenosyl-transferase), in which a high [GSH]/[GSSG] (glutathione disulfide) ratio promotes SAM synthase to achieve its maximum activity (Pajares et al., 1992; Rahman et al., 2003; Hitchler and Domann, 2007). During oxidative stress, Ergothioneine interacts with the KEAP1–NRF2 signaling pathway, and NRF2 subsequently upregulates a multitude of antioxidation genes in the GSH-based system, including γ-glutamate cysteine ligase catalytic subunit (γ-GCLC), γ-glutamate cysteine ligase modifier subunit (γ-GCLM), glutathione reductase, glutathione peroxidase and glutathione *S*-transferase α2 (GSTA2) (Hayes and Dinkova-Kostova, 2014). Ergothioneine in the antioxidant system may therefore influence the cellular GSH amount and [GSH]/[GSSG] ratio, and those effects could further transmit to the availability of SAM, preventing the dysregulation of cellular methylation function. Additionally, as reviewed by Garcia-Gimenez and Pallardo (2014) and Garcia-Gimenez et al. (2014), GSH may influence epigenetic mechanisms in more ways than just regulating SAM levels. Perhaps Ergothioneine and GSH metabolic associations may lead to more complex epigenetic regulations, especially in redox imbalanced senescent cells.

#### Epigenetic demethylation

In addition to epigenetic methyltransferases, demethylases such as the ten–eleven translocation family demethylases (TETs) may also be influenced by Ergothioneine. The TETs are non-heme iron (NHFe) dependent monooxygenases, whose activities are severely affected by the availability of iron in the labile iron pool (Kakhlon and Cabantchik, 2002; Camarena et al., 2021). The abundance and oxidation state of cellular free irons are seriously affected by ROS, meanwhile, free ferrous (Fe^II^) and ferric (Fe^III^) play critical roles in the development of fairly-benign ROS species into more toxic ones. Under oxidative stress, superoxide radicals release ferrous ions from certain iron-sulfur cluster proteins, ferritins, and transferrins. Furthermore, excessive hydrogen peroxides decompose heme and release free irons from heme proteins, including myoglobin, hemoglobin and cytochrome *c* (Borodina et al., 2020; Halliwell, 2020). The unliganded irons promote the formation of hydroxyl radicals from superoxide radicals and hydrogen peroxides via iron-catalyzed Haber–Weiss reactions (reaction 1). Reaction (1) is the sum of reactions (2) and (3), in which reaction (2) is also known as the Fenton reaction. The hypochlorous acid equivalent could be written as reaction (4) (Wardman and Candeias, 1996; Kehrer, 2000).


$$\begin{array}{l} O_{2}^{\;\cdot \;-}+H_{2}O_{2}\to \;\cdot OH+OH^{-}+O_{2} \end{array}$$ (1)

$$\begin{array}{l} Fe^{II}+H_{2}O_{2}\to Fe^{III}+\cdot OH+OH^{-} \end{array}$$ (2)

$$\begin{array}{l} Fe^{III}+O_{2}^{\;\cdot \;-}\to Fe^{II}+O_{2} \end{array}$$ (3)

$$\begin{array}{l} Fe^{II}+HOCl\to Fe^{III}+\cdot OH+Cl^{-} \end{array}$$ (4)

These iron-dependent ROS autoxidation reactions produce highly toxic hydroxyl radicals, which can quickly damage the biomacromolecules. To prevent such reactions, cells sequestrate transition metal ions, especially iron (Halliwell, 2020). The ROS-induced iron restriction had been supported by the observation that exogenous H_2_O_2_ attenuated the demethylation activity of TET demethylases and resulted in an epigenetic shift (Niu et al., 2015; Hitchler and Domann, 2021). Ergothioneine’s remarkable ability to rapidly neutralize hydroxyl radicals makes it a critical player in the maintenance of cellular redox homeostasis. Other ROS, such as hydrogen peroxide, superoxide and hypochlorites, could also be targeted by Ergothioneine, in the manner of both a self-sacrificing antioxidant and a regulator of cellular antioxidant defense system (Figs. 2 and 3). Therefore, cellular iron co-factors may indirectly but critically be affected by Ergothioneine.

Similar to DNA, cellular RNA and histone are also decorated with chemical modifications, and such modifications participate in many aspects of life processes, including ageing (Michalak et al., 2019). Methylation/demethylation of RNA and histone could also be affected by Ergothioneine because both the histone methyltransferases (HMTs) and methyltransferase-like proteins (METTLs) employ SAM as a methyl group donor (Fig. 2), as well as the Jumonjic (JmjC)-domain-containing histone demethylase and the m^6^A demethylase (FTO, AlkBs) are NHFe/α-KG-dependent dioxygenases (Shi, 2007; Jia et al., 2011; Markolovic et al., 2016). Collectively, the epigenetic modifications to DNA, RNA, and histone would be greatly affected by varying the access to epigenetic enzyme co-factors, such as SAM and ferrous, that may be influenced by Ergothioneine. Although the direct control of Ergothioneine in epigenetic regulation has not been revealed, it would make sense that Ergothioneine preserves cellular redox balance to influence the availability of SAM and ferrous, ultimately preventing epigenetic alterations. Further investigations are required to elucidate whether and how Ergothioneine impacts epigenetic modifications with ageing.

### Ergothioneine regulates the Sirtuin pathways

Beyond the methylation/demethylation modifications on DNA, RNA, and histone, acetylation/deacetylation of histone plays important roles in cellular ageing. Sirtuins, a family of NAD^+^-dependent deacetylases, play critical roles in a range of biological processes, including but not limited to epigenetic reprogramming and epigenetic drift, DNA damage repair and genome stability, oxidative stress and antioxidant defense pathways, mitochondrial function, as well as healthy longevity (Finkel et al., 2009; Singh et al., 2018). In mammals, the Sirtuin family of seven enzymes has been linked to both epigenetic functions and metabolic regulation (Brunet et al., 2004; Chen et al., 2005; Kawahara et al., 2009; Kanfi et al., 2012). Several findings indicated that SIRT1 and SIRT6 provide both profound health benefits and potent longevity activities (Kanfi et al., 2010, 2012; Satoh et al., 2013).

Ergothioneine interacts with the Sirtuin pathways to regulate ageing. It has been reported that Ergothioneine protects against endothelial senescence by regulating a group of Sirtuins. A study by D’Onofrio et al., revealed that Ergothioneine protects endothelial cells against high-glucose treatment through the upregulation of SIRT1 and SIRT6. Additionally, Ergothioneine’s protective effect against endothelial senescence was reduced when SIRT1 activity is inhibited or the SIRT6 gene is silenced (D’onofrio et al., 2016). Another recent study from the same group showed that Ergothioneine induces necroptosis in colorectal cancer cells by upregulating SIRT6 (D’onofrio et al., 2022). Both of the studies revealed that Ergothioneine exerts anti-ageing and anti-cancer properties via Sirtuin signaling, suggesting that Ergothioneine has a dynamic regulatory role in ageing signaling pathways.

These findings, combined with the fact that Sirtuin’s deacetylase consumes NAD^+^, an essential redox signaling molecule, reminiscing the idea that Ergothioneine may affect Sirtuins’ activity through modulating NAD^+^ availability. [NAD^+^]/[NADH] ratio is a key redox indicator of the metabolic and physiological status of the cell (Imai and Guarente, 2014), and NAD^+^ depletion caused by oxidative stress is harmful for the proper functioning of Sirtuins. As an antioxidant, Ergothioneine could control the prooxidant-antioxidant balance and maintain the metabolic co-factor pool of NAD^+^, which affects Sirtuins’ function directly. In addition, as summarized by Kalous et al. (2021), one of the mechanisms that decrease the activity of Sirtuins could be the oxidative post-translational modification by ROS and RNS. In ageing cells, ROS and RNS increase, contributing to the loss of Sirtuin activity (Salminen et al., 2013). Ergothioneine, the scavenger of ROS and RNS, might also enhance Sirtuins’ activity by preventing oxidative modifications to Sirtuin enzymes.

In mammals, oxidative stress and epigenetic functions are closely interconnected (Guillaumet-Adkins et al., 2017). SIRT1 is the main deacetylase of histones H3 and H4, as well as a direct regulator of SUV39H1 methyltransferase, promoting SUV39H1 activity on H3K9 methylation (Fig. 3) (Jing and Lin, 2015). Oxidative stress in rat myocytes was shown to induce a rapid upregulation of SUV39H1 (Yang et al., 2017). Additionally, a genome-wide distribution study of histone marks showed that during oligodendrocyte differentiation, a large portion of H3K9Me3 modifications can be mapped to the gene body encoding functional proteins (Liu et al., 2015). These data suggest that SIRT1 and SUV39H1 may involve in euchromatin transcription regulation in distinct tissues when exposed to oxidative stress. In a 2022 review (Padeken et al., 2022), Padeken highlighted emerging evidence that H3K9me3 is upregulated by oxidative stress and proposed that the alternation of epigenetic landscape in specific tissues may result from a long-term adaptation to stress. With Ergothioneine’s significant contribution to antioxidative stress, it is an intriguing possibility that Ergothioneine’s anti-ageing effect could be partially attribute to its impact on epigenetic dynamics. Collectively, the beneficial effects of Ergothioneine on cellular epigenetics, metabolism, and ageing could be mediated through Sirtuin pathways, although the details remain to be thoroughly explored.

Studies have also revealed a close linkage between Sirtuins and the KEAP1–NRF2 pathways. NRF2 is one of the common targets for Sirtuins in the regulation of antioxidative genes (Pan et al., 2016; Xue et al., 2016; Singh et al., 2018). SIRT1 is known to deacetylate NRF2 and contribute to its stability as well as stimulate the transport of NRF2 to the nucleus (Huang et al., 2013; Yang et al., 2014; Singh and Ubaid, 2020). In addition to enhancing NRF2 transcription and translocation, SIRT1 also negatively impacts its polyubiquitination by reducing the expression of KEAP1/CUL3, as well as increasing the binding ability of NRF2 to ARE (Wang et al., 2019). Moreover, SIRT6 positively regulates the NRF2-ARE antioxidant pathway as a NRF2 coactivator. It reduces the acetylation level of H3K56 to facilitate chromatin looping. As a scaffold, SIRT6 recruits RNA polymerase II (RNAP II) to generate a NRF2–SIRT6–RNAP II complex, leading to transcriptional activation of NRF2-regulated antioxidant genes (Pan et al., 2016; Rezazadeh et al., 2019). Ergothioneine affects both Sirtuins and the KEAP1–NRF2 pathways, supporting the hypothesis that it is not only an antioxidant but also an anti-ageing agent.

During the last 2 decades, Ergothioneine has attracted considerable attention due to its potential as an antioxidant and anti-ageing compound to treat numerous age-related ailments and even extend lifespan. Ergothioneine is believed to modulate the level of epigenetic enzyme Sirtuins, as well as the supplementation of epigenetic enzyme co-factors, including ferrous ions, SAM, and NAD^+^, leading to an Ergothioneine–Epigenome–Longevity axis. Since the epigenetic dynamics play one of the most significant roles in development, ageing, disease, and longevity, it is important to explore the effects of Ergothioneine on epigenome toward histone acetylome, as well as DNA, RNA, and histone methylome. Uncovering the functional mechanism of Ergothioneine would be a challenging and invaluable task, and it would lead to great advances in the development of Ergothioneine as an anti-ageing agent.

## Biosynthesis of Ergothioneine, Ovothiol, and Selenoneine

Accumulated discoveries have bolstered the evidence of Ergothioneine’s anti-ageing activity and its therapeutic potential against ageing-associated diseases, leading to further exploration into its biosynthesis and that of related compounds. Trans-sulfur reactions involved in Ergothioneine and Ovothiol biosynthesis, as well as the unique selenium metabolic pathway responsible for Selenoneine production, have attracted broad attention in the field of natural product biosynthesis and synthetic biology. This section summarizes the pharmacophore formation steps, particularly the enzymatic mechanism of C–S bond formation reactions.

### Ergothioneine biosynthesis

**Figure 4.**
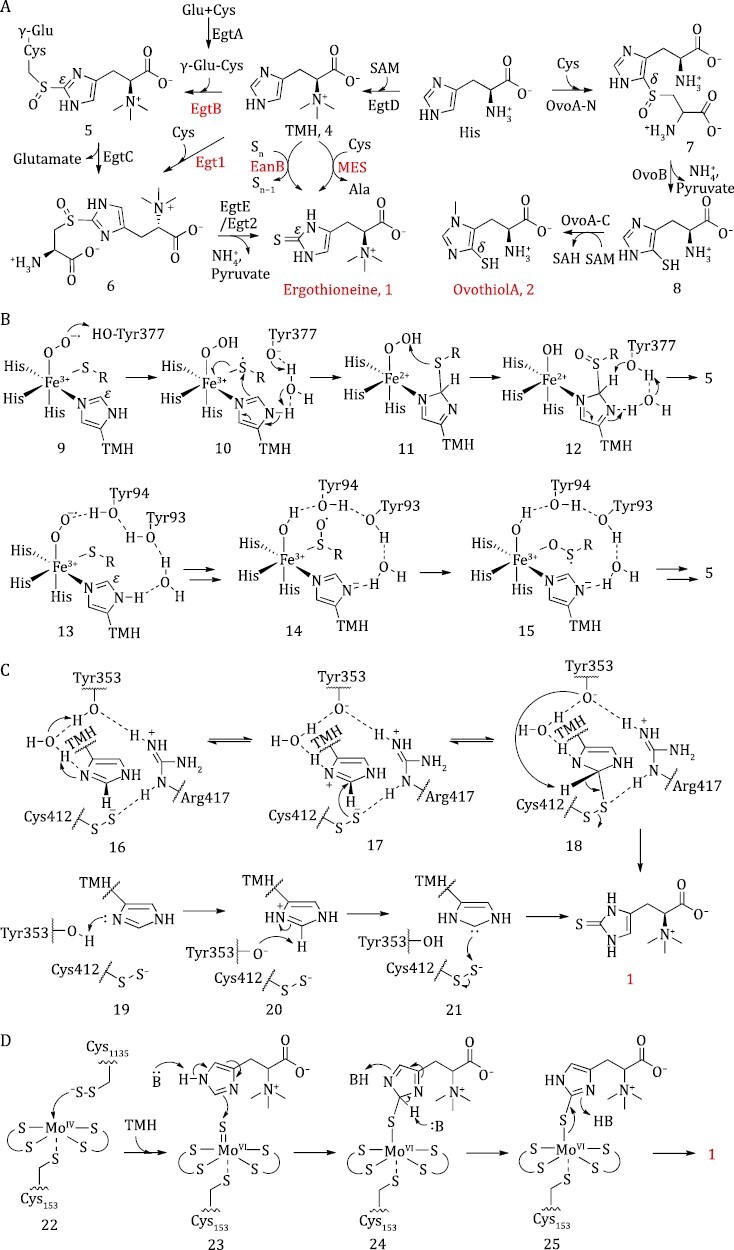
**Biosynthesis of Ergothioneine and Ovothiol A**. (A) Ergothioneine and Ovothiol biosynthetic pathways. (B) *Mth*EgtB (top row) and *Cth*EgtB (bottom row) enzymatic proposals; (C) EanB proposals from Seebeck’s model (top row) and Liu’s model (bottom row); (D) MES proposal.

For over 100 years, Ergothioneine has been known to process amazing biological activities, yet it was only in the last few decades that its biosynthetic pathway has been revealed. Generally, there are a few distinct pathways for Ergothioneine synthesis in nature: the most common mechanism includes the oxygen-dependent formation of C–S bond, catalyzed by iron-dependent sulfoxide synthases (EgtB and Egt1) (Seebeck, 2010; Hu et al., 2014). The alternative pathways involve oxygen-independent sulfur transformations, catalyzed by Ergothioneine synthases (EanB and MES) (Fig. 4A) (Burn et al., 2017; Beliaeva and Seebeck, 2022). In the first step of EgtB-pathway, methyltransferase EgtD catalyzes the trimethylation of histidine to form trimethylhistidine (TMH, 4) using SAM as methyl donors. The following step is then catalyzed by EgtB, coupling TMH with γ-Glu-Cys (γ-GC, which is formed by ligating glutamic acid and cysteine through EgtA). EgtC and EgtE then trim the sulfoxide intermediate (5) to eventually release Ergothioneine (Seebeck, 2010). A similar but simplified pathway was catalyzed by Egt1&2: cysteine is used as the sulfur-donor directly, Egt1 catalyzes sulfoxide formation and Egt2 assumes the functions of EgtE to generate Ergothioneine (Hu et al., 2014). On the other hand, the oxygen-independent EanB- and MES-pathways eschew sulfoxide intermediates, instead, the C–S bonds are formed by transferring sulfur from polysulfides (“S_n_”) or cysteine persulfides (CysSSH) to TMH directly (Burn et al., 2017; Beliaeva and Seebeck, 2022).

#### Oxygen-dependent C–S bond formation

The crystal structures of two sulfoxide synthases, *Mth*EgtB from *Mycobacterium thermoresistibile* and *Cth*EgtB from *Chloracidobacterium thermophilum*, which prefer γ-Glu-Cys and cysteine as sulfur-donors, have been resolved, respectively (Goncharenko et al., 2015; Naowarojna et al., 2019; Stampfli et al., 2019). *Mth*EgtB and *Mth*EgtB-substrate complex structures showed that the catalytic Fe^II^ is coordinated by three histidine residues in a facial geometry, and the three remaining coordination sites of Fe^II^ center are occupied by the sulfur-donor γ-GC, the sulfur acceptor TMH and a crystallographic water. Mutagenesis and kinetic experiments of *Mth*EgtB showed that a conserved residue Tyr377 plays a critical role in C–S bond formation and sulfoxidation (Fig. 4B). Replacement of Tyr377 with phenylalanine dramatically reduced the sulfoxide synthase activity of EgtB while remained its side activity as a cysteine dioxygenase (Goncharenko and Seebeck, 2016).

Several mechanistic models have been proposed for *Mth*EgtB on the basis of computational studies, which all stem from the reactive Fe^III^–superoxo complex: a generally believed intermediate in many non-heme iron oxidases formed by oxidative addition of O_2_ to the Fe^II^ center (Faponle et al., 2017; Wei et al., 2017; Tian et al., 2018). Three points have been raised with distinct opinions: (i) whether thioether formation (C_ε_–S bond formation) or sulfenic acid formation (hydroxylation of the sulfur atom) is the first half of EgtB reaction; (ii) Whether the C_ε_–H cleavage occurs before or after the C_ε_–S bond formation? (iii) Is the active site tyrosine Tyr377 a redox agent or an acid–base catalyst? The Liao and Liu models suggest that hydroxylation of the sulfur atom is the first half, while the Visser model prefers that C_ε_–S bond formation is the first one. The catalytic residue Tyr377 was proposed to play a redox-active role in the Visser model, but was not involved in the Liu model. Additionally, the Liao and Liu models have also predicted that C_ε_–H cleavage is the rate determining step, at least in part, and *R*-sulfoxide is the reaction product, yet experimental observations indicate a close to unity substrate KIE and *S*-sulfoxide product formation (Goncharenko and Seebeck, 2016).

In a recent review, Stampfli and Seebeck combined computational studies and experimental work to develop a *Mth*EgtB proposal, suggesting that protonation of Fe^III^-superoxo (9) by Tyr377 induces a thiyl radical of γ-GC, and deprotonation of the TMH imidazole ring initiates its attack to the electron-deficient thiyl radical (10). C_ε_–S bond formation precedes hydroxylation of the sulfur atom (11), and heterolytic cleavage of the C_ε_–H bond (12) gives the sulfoxide intermediate (5) (Fig. 4B) (Goncharenko et al., 2020; Stampfli and Seebeck, 2020). It partially aligns with the Visser computational model, the main difference is that Tyr377 functions as a Lewis acid–base but not the redox agent (Faponle et al., 2017).

The overall structure of *Cth*EgtB is similar to that of *Mth*EgtB with a difference that there are two essential tyrosine residues, Tyr93 and Tyr94, in the active center. Computational study based on *Cth*EgtB crystal structure has been carried out as well, and results indicate that *Cth*EgtB Tyr93–Tyr94 is the counterpart of *Mth*EgtB Tyr377, functioning as an acid-base catalyst (13). Hydroxylation of the sulfur atom occurred prior to C_ε_–S bond formation, C_ε_–H cleavage is no longer the rate determining step. Notably, a coordination switch of γ-GC sulfenic acid intermediate from sulfur (14) to oxygen atom (15) was proposed (Wu et al., 2022; Zhang et al., 2023). It well accommodates the EgtB experimental results, and sheds light on the mechanism of other sulfoxide synthases, including Egt1 and OvoA (C_δ_–S bond formation enzyme involved in Ovothiol biosynthesis). Although significant progresses have been achieved in the study of EgtB chemistry, the EgtB catalytic mechanism is still not well understood. Trapping and characterizing the intermediates are needed to clarify the debates about the reaction sequence and the exact role of active site tyrosine residue.

#### Oxygen-independent sulfur transformation

The anaerobic biosynthetic pathways of Ergothioneine were revealed recently. Oxygen-independent Ergothioneine synthase EanB from the green sulfur bacterium *Chlorobium limicola* has been characterized, and the enzymatic mechanism was proposed on the basis of protein crystal structure and kinetic studies (Fig. 4C) (Burn et al., 2017; Leisinger et al., 2019). Active site residues Tyr353 and Cys412 were found to play critical roles in the TMH-sulfurization-reaction. Tyr353 protonates the imidazole ring of TMH (16) at first, then the nucleophilic cysteine persulfide anion (Cys412-SS^−^) attacks the imidazole ring to form C_ε_–S bond (17), and deprotonation of the imidazole ring by Tyr353 base (18) releases Ergothioneine ultimately. Another independent work further identified that polysulfide could be used as sulfur source directly (Cheng et al., 2020, 2021). It has reached a consensus that protonation (19) and deprotonation (20) of the TMH imidazole ring by Tyr353 are key steps of EanB catalysis, but the C_ε_–S bond is formed via an imidazole-2-yl carbene intermediate (21) in the Liu model on the basis of detection of an ε-carbon deuterium/hydrogen exchange reaction in EanB catalysis and quantum mechanics/molecular mechanics calculations. Computational studies showed that the carbene pathway is energetically preferable and the Arg417 guanidinium group and Tyr353 phenol group play key roles in the stabilization of carbene intermediates (Cheng et al., 2021; Lai and Cui, 2022).

A very recent work based on genome mining identified another oxygen-independent enzyme MES for Ergothioneine synthesis from the anaerobic bacterium *Caldithrix abyssi* (Beliaeva and Seebeck, 2022). MES harbors two functional domains, in which the C-terminal one is a cysteine desulfurase and the N-terminal one is a metallopterin-dependent enzyme responsible for the sulfurization of TMH. It is distinct from oxygen-dependent sulfoxide synthases EgtB, Egt1, and OvoA, which are iron-dependent enzymes. MES desulfurizes free cysteine and then transfers the sulfur onto TMH with Cys1074 and Cys1135 as intramolecular sulfur-transport-chain in form of cysteine persulfide CysSSH. For the C_ε_–S bond formation, sulfur is transferred from protein-borne CysSSH to the Mo^IV^ co-factor (22), generating an active Mo^VI^ = S species (23), then a nucleophilic attack on the sulfido ligand of Mo^VI^ = S by TMH imidazole ring initiates the reaction. Base assisted intermediate tautomerization (24) and product dissociation (25) recycle the co-factor to a reduced state, which is ready for the next round of trans-sulfur reaction. This is the first example that the mononuclear molybdenum dependent enzyme catalyzes carbon-sulfur bond formation.

### Ovothiol biosynthesis

Ovothiol, a homologue of Ergothioneine, is another histidine derivative containing sulfur substitutions at δ position carbon of the imidazole ring. Found in a variety of marine invertebrates, algae, and protozoa, it plays a significant role in protecting against oxidative stress in the fertilization and embryo-release processes in seawater. It shows antioxidant properties and amazing potential to treat chronic low-grade systemic inflammation and related diseases (Castellano and Seebeck, 2018; Brancaccio et al., 2022). Three types of ovothiols have been characterized, with Ovothiol A’s α-amino group being unmethylated, while B and C are respectively mono- and di-methylated. The biosynthetic pathway of Ovothiol A has been established and the trans-sulfur reaction was then deciphered. The N-terminal domain of OvoA, a homolog of EgtB and Egt1, catalyzes the coupling of cysteine and histidine to give a sulfoxide intermediate (7) (Braunshausen and Seebeck, 2011). The sulfoxide is then cleaved by OvoB to release pyruvate and ammonia, with the C-terminal domain of OvoA methylating the π-nitrogen of imidazole ring to produce Ovothiol A (Fig. 4A) (Naowarojna et al., 2018).

Although OvoA’s crystal structure has not been determined, homology modeling and site-directed mutagenesis studies have shown that Tyr417 in OvoA from *Erwinia tasmaniensis* (*Eta*OvoA) is the counterpart of Tyr377 in *Mth*EgtB. To explore the role of Tyr417, an unnatural amino acid 2-amino-3-(4-hydroxy-3-(methoxyl) phenyl) propanoic acid (MeOTyr), a tyrosine analogue which has comparable p*Ka*, but a ~200 mV lower reduction potential relative to that of tyrosine, has been incorporated into *Eta*OvoA. Analyses of the OvoA Y417MtTyr variant reaction with [*U*-^2^H_5_]-Histidine revealed a deuterium isotope effect of 0.86 ± 0.03 (Chen et al., 2019), which is consistent with the computational prediction of an inverse isotope effect (Faponle et al., 2017). Meanwhile, the wild type *Eta*OvoA enzyme reaction with [*U*-^2^H_5_]-his yielded a substrate KIE of 1.01 ± 0.02, departing from the large magnitude KIE (5.7) predicted by another computational study about EgtB (Wei et al., 2017; Chen et al., 2018). Collectively, our experimental results prefer the Visser model that active site Y417 is part of a proton-coupled electron transfer process, C_ε_–S bond formation precedes C_ε_–H homolytic cleavage (Faponle et al., 2017; Chen et al., 2018, 2019). This is not fully consistent with the *Mth*EgtB enzymatic proposal, in which the corresponding tyrosine residue (Y377) functions as a Lewis base (Stampfli and Seebeck, 2020). It is important to note that even though EgtB and OvoA are similar, they are not identical, since they show distinct substrate preference and regioselectivity. Further, a recent study on *Methyloversatilis thermotolerans* OvoA (*Mth*OvoA) has indicated that its sulfoxide synthetase activity is still maintained when the tyrosine residue at the active site was mutated to phenylalanine (Cheng et al., 2022), indicating that there might be certain overlooked interactions that direct the product formation in the reaction serials of sulfoxide synthesis.

### Selenoneine biosynthesis

**Figure 5.**
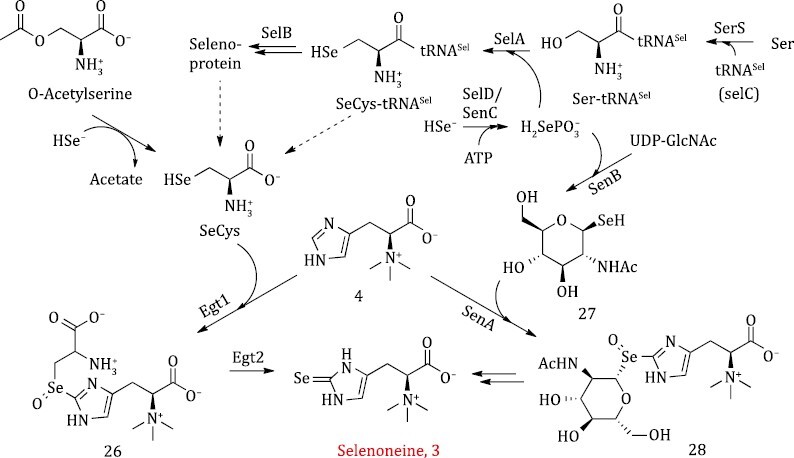
**Biosynthetic pathway of Selenoneine**.

Selenoneine, discovered from the blood of bluefin tuna and the culture broth of fission yeast *Schizosaccharomyces pombe,* is another homologue of Ergothioneine, featuring a selenium atom substitutedfor the sulfur atom. Compared to Ergothioneine, Selenoneine exhibits almost 1,000-fold stronger radical-scavenging ability (Yamashita and Yamashita, 2010; Pluskal et al., 2014). The source of Selenoneine in tuna cells remains inconclusive: it is uncertain whether Selenoneine is produced by tuna cells or if it is an exogenous nutrient enriched in their blood cells. Seneloneine could be generated from a divergent biosynthetic pathway of Ergothioneine in the environment of high Selenocysteine (SeCys) concentration. Introduction of *S. pombe egt1* and *egt2* genes into *Aspergillus* established an artificial pathway for Seneloneine biosynthesis (Fig. 5) (Pluskal et al., 2014; Turrini et al., 2018), and a hercynylselenocysteine intermediate (26) was proposed (Goncharenko et al., 2020). This suggests that SeCys may act as the selenium donor for Selenoneine synthesis. Free SeCys emerge from two distinct routes, one of which is seleno-protein ribosomal assembly line, and the other is mis-incorporation of selenium into the cysteine *de novo* biosynthetic pathway. To protect against oxidative stress and the mis-incorporation of selenium in ribosomal translation, cellular concentration of SeCys is limited, thereby explaining why both *S. pombe* and recombinant *Aspergillus* produce very little Selenoneine despite being exposed to exogenous selenates in their culture media.

Recently, a Selenoneine biosynthetic pathway was identified in *Variovorax paradoxus* that involves *N*-acetyl-1-seleno-β-d-glucosamine (SeGlcNAc) as the direct selenium-donor (Fig. 5) (Kayrouz et al., 2022). SeGlcNAc is synthesized by similar initial steps with that of SeCys-tRNA^Sel^, which is a building block of seleno-protein. HSe^-^ is activated by ATP, forming H_2_SePO_3_^−^ through the catalysis of SelD (also named as SenC in Selenoneine pathway), then H_2_SePO_3_^−^ is used to generate SeCys-tRNA^Sel^ under the catalysis of SelA in seleno-protein assembly (Stadtman, 1996). Alternatively, H_2_SePO_3_^−^ could be used to form SeGlcNAc (27) under the catalysis of SenB for Selenoneine biosynthesis. Similar to the hercynylcysteine sulfoxide intermediate involved in Ergothioneine biosynthesis, a hercynyl-SeGlcNAc selenoxide intermediate (28) formed by SenA has been identified in Selenoneine biosynthesis. This intermediate spontaneously fragmentates to generate Selenoneine, or is reduced by reductants such as thiols and ascorbate to give a selenoether (Goncharenko et al., 2020). Comparing to the Ergothioneine-divergent pathway, the novel enzyme SenA provides an effective rout for Selenoneine formation. This work filled the gap in our understanding of Seneloneine biosynthesis and expanded our knowledge of selenium metabolism.

## Concluding remarks

Ergothioneine has been widely recognized to possess therapeutic potential in a variety of ageing-associated diseases, and promoting healthy longevity of humans. According to the free radical theory of ageing, the production of intracellular ROS is the major driving forces of ageing (Balaban et al., 2005). Ergothioneine-mediated ROS elimination has the potential to mitigate genomic instability and epigenetic alterations, two of the hallmarks of ageing. It is suggested that Ergothioneine has the capacity to regulate antioxidant defense KEAP1–NRF2 pathway, interact with Sirtuin-mediated epigenetic pathways, and influence epigenetic methylation/demethylation. Taken together, we suppose that Ergothioneine is more of a regulatory factor than a self-sacrificing antioxidant, and a possible Ergothioneine–Epigenome–Longevity axis was consequently proposed. The excellent biological activities of Ergothioneine, Ovothiol, and Seneloneine have prompted substantial study attention to the elucidation of their biosynthetic pathways. Detailed enzymatic mechanisms of key steps in these pathways, especially the pharmacophore-forming reactions, have been deciphered extensively. The oxygen-dependent sulfoxide synthases, EgtB, Egt1, and OvoA represent a novel sulfurtransferase branch distinct from the well-known Rhodanese, and SenA for C–Se bond formation have been identified and characterized, expanding our understanding in sulfur and selenium utilization in nature. The oxygen-independent Ergothioneine synthases, EanB and MES, exhibit two unprecedented biochemical reactions. These achievements pave the way for the development of metabolic engineering and synthetic biotechnologies for industrial production of Ergothioneine, Ovothiol, and Seneloneine.

## Abbreviations

5mC: C5 methylation of cytosine
γ-GC: γ-Glu-Cys
γ-GCLC: γ-glutamate cysteine ligase catalytic subunit
γ-GCLM: γ-glutamate cysteine ligase modifier subunit
ABC: ATP-binding cassette
AKT: serine/threonine kinase
ARE: antioxidant response element
ATP: adenosine 5ʹ-triphosphate
CAT: catalase
ClO^−^: hypochlorite
CUL3: Cullin3
CysSSH: cysteine persulfides
DNMTs: DNA methyltransferases
DRACH: motif sequence, D = G/A/U, R = G/A, H = A/U/C
EGT: ergothioneine
FTO: fat mass and obesity associated protein
GSH: glutathione
GSSG: glutathione disulfide
GSTA2: glutathione *S*-transferase α2
HMTs: histone methyltransferases
HO-1: heme oxygenase-1
H1K26: histone 1 lysine 26
H3K9: histone 3 lysine 9
H3K14: histone 3 lysine 14
H3K56: histone 3 lysine 56
H3K9Me3: histone 3 with trimethylated lysine 9
H4K6: histone 4 lysine 6
JmjC: JumonjiC
KEAP1: Kelch-like ECH-associated protein 1
KIE: kinetic isotope effect
MeOTyr: 2-amino-3-(4-hydroxy-3-(methoxyl) phenyl) propanoic acid
METTLs: methyltransferase-like proteins
mtDNA: mitochondrial DNA
NAD+: nicotine adenine dinucleotide
NHFe: non-heme iron
NQO1: NAD(P)H, quinone oxidoreductase
NRF2: nuclear factor erythroid 2-related factor 2
^1^O_2_: singlet oxygen
O_2_^·−^: superoxide radical
H_2_O_2_: hydrogen peroxide
OH: hydroxyl radical
OCTN1: organic cation transporter 1
ONOO^−^: peroxynitrite
PI3K: phosphatidylinositol 3-kinase
PKC: protein kinase C
RNAP II: RNA polymerase II
RNS: reactive nitrogen species
ROS: reactive oxygen species
SAM: *S*-adenosylmethionine
SeCys: selenocysteine
sMAF: small Maf protein
SeGlcNAc: *N*-acetyl-1-seleno-β-d-glucosamine
SIRTs: sirtuins
SIRT1: sirtuin 1
SIRT6: sirtuin 6
SLC22A4: solute carrier family 22 member 4
SOD: superoxide dismutase
SUV39H1: suppressor of variegation 3–9 homolog 1
TF: transcription factor
TMH: trimethylhistidine
TETs: ten–eleven translocation family demethylases
Ub: ubiquitin
UV/IR: ultraviolet/infrared
